# BNP on Admission Combined with Imaging Markers of Multimodal CT to Predict the Risk of Cardioembolic Stroke

**DOI:** 10.1155/2022/3327967

**Published:** 2022-07-26

**Authors:** Ruoyao Cao, Yun Jiang, Ling Li, Yao Lu, Junjie Wang, Kezhen Yu, Min Chen, Juan Chen

**Affiliations:** ^1^Department of Radiology, Beijing Hospital, National Center of Gerontology, Institute of Geriatric Medicine, Chinese Academy of Medical Sciences, China; ^2^Graduate School of Peking Union Medical College, Beijing, China; ^3^Department of Neurology, Beijing Hospital, National Center of Gerontology, Institute of Geriatric Medicine, Chinese Academy of Medical Sciences, China; ^4^Department of Neurosurgery, Beijing Hospital, National Center of Gerontology, Institute of Geriatric Medicine, Chinese Academy of Medical Sciences, China

## Abstract

**Background:**

The aim of the study was to find the potential roles of B-type natriuretic peptide (BNP) and imaging markers on distinguishing cardioembolic (CE) stroke from non-CE stroke, so as to provide useful information for making individualized endovascular treatment (EVT) plan for the patients with acute ischemic stroke (AIS).

**Methods:**

The patients with unilateral anterior circulation large vessel occlusion who underwent EVT between March 2016 and December 2021 were analyzed in this study, retrospectively. The risk factors, laboratory test indicators, imaging parameters, and other factors were compared between the CE group and non-CE group. Logistic regression was used to analyze the risk factors of CE stroke. ROC curves were used to assess the values of different parameters on distinguishing CE stroke from non-CE stroke. The relationships between BNP and imaging parameters were assessed using the Spearman correlation analysis.

**Results:**

160 patients were enrolled in the study and divided into the CE group (*n* = 66) and non-CE group (*n* = 94). BNP (odds ratio (OR) = 1.004; 95% CI, 1.001-1.009; *p* = 0.038), MMR (OR = 0.736; 95% CI, 0.573-0.945; *p* = 0.016), NIHSS (OR = 1.150; 95% CI, 1.022-1.294; *p* = 0.020), and AF (OR = 556.968; 95% CI, 51.739-5995.765; *p* < 0.001) were the independent predictive factors of CE stroke. The area under the curve (AUC) of BNP and mismatch ratio (MMR) were 0.846 (95% CI (0.780-0.898), *p* < 0.001) and 0.636 (95% CI (0.633-0.779), *p* < 0.001), respectively. The cut-off value of BNP was 249.23 pg/mL with the sensitivity of 74.24% and the specificity of 82.98%. BNP combined with MMR improved the predictive value for CE stroke. The AUC of the combination was 0.858 with the sensitivity of 84.85% and the specificity of 73.40%. BNP was correlated with 4D CTA collateral score, MMR, clot burden score, final infarct volume, infarct core volume, and ischemic penumbra volume (all, *p* < 0.05).

**Conclusion:**

BNP on admission combined with MMR is valuable for the risk prediction of CE stroke, which will promote the further screening of the high-risk patients with CE stroke and provide more diagnostic information for clinicians.

## 1. Introduction

Endovascular treatment (EVT) has emerged as a standard treatment for patients with acute ischemic stroke (AIS) [1, 2]. However, different stroke subtypes show different therapeutic efficacies of EVT, as well as different prognosis and risks of postoperative complications. Patients with cardioembolic (CE) stroke have more serious neurological deficits and the worse clinical prognosis [3, 4]. Moreover, difficulties remain in diagnosis of CE stroke and there is no a unified diagnostic standard for CE stroke. Some studies have tried to identify blood biomarkers to assist in the rapid screening of CE stroke, thereby formulating timely diagnosis and treatment schemes [5, 6]. Among them, B-type brain natriuretic peptide (BNP) is a research hotspot, but with many controversies [5, 7–10]. However, due to the complexity of the pathogenesis of stroke, the diagnosis cannot be confirmed by a single blood biomarker.

In recent years, several randomized clinical controlled trials on EVT have applied multimodal imaging techniques to strictly screen the AIS patients [1, 2, 11, 12]. These imaging techniques help doctors to exclude hemorrhage, identify the occluded vessels, and evaluate the infarct core (IC) volume, ischemic penumbra (IP) volume, and collateral circulation status, which may provide abundant and effective information for making clinical decisions [13]. For the clinical diagnosis and treatment of stroke subtypes, it is of critical importance to explore valuable imaging markers and evaluate them together with blood markers.

Herein, this study intends to find the potential roles of BNP and imaging markers on distinguishing CE stroke from non-CE stroke, with the aims to provide useful information for making the individualized EVT plan of AIS patients.

## 2. Methods

### 2.1. Study Population

Suspected AIS patients with anterior circulation large vessel occlusion who underwent EVT in our center from March 2016 to December 2021 were retrospectively reviewed. All the patients performed multimodal CT (including noncontrast CT, 4D CT angiography (4D CTA), and CT perfusion (CTP)) before treatment. An attending neurosurgeon as well as a neurologist and a neuroradiologist made the treatment decision. The Ethics Committee of the Beijing Hospital approved the study (No. 2020BJYYEC-267-01) and waiving written informed consent because the data were retrospectively and anonymously evaluated.

**Figure 1:**
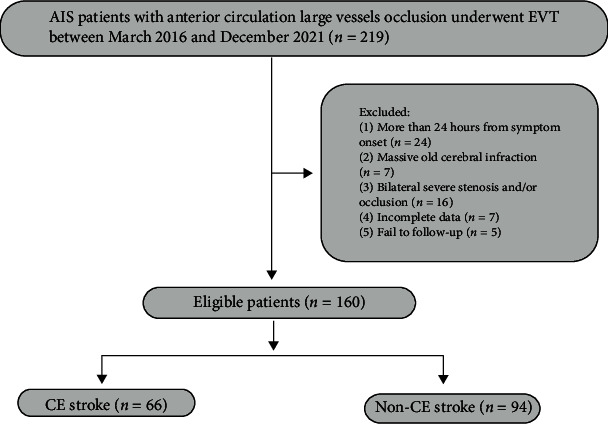
Flowchart of patient selection.

Exclusion criteria are as follows: (1) hemorrhagic stroke; (2) more than 24 hours from symptom onset; (3) massive old cerebral infarction; (4) bilateral severe stenosis and/or occlusion; and (5) incomplete clinical, laboratory, or imaging data (Figure 1).

### 2.2. Data Collection

#### 2.2.1. Imaging Protocol

All images were acquired with a 320 × 0.5 mm detector rows CT (Aquilion ONE, Canon Medical Systems). 40-50 mL nonionic iodine contrast agent (iopamidol, Bracco, Shanghai, China) was intravenously injected with the dose of 0.6 mL/kg, followed by 30 mL saline. NCCT scan parameters are 135 kV and 300 mAs. 4D CTA-CTP scan parameters are 80 kV and 100 mAs.

ASPECTS [14] was used to assess early ischemic changes. Vitrea (Vital Images, Minnetonka, Minnesota) was utilized for obtaining CTP parameters, including cerebral blood volume (CBV) and time to peak (TTP). IC was defined as the area with a 38% decrease in CBV as well as a 5.3-second increase in TTP, while IP was defined as the area with a 5.3-second increase in TTP, without a decrease in CBV. Mismatch ratio (MMR) was calculated by dividing the IP volume by the IC volume [15]. The modified collateral circulation scoring system on 4D CTA (4D CTA-CS), a 5-point scale grading system, was used to assess the collateral status [16]. Colt burden score (CBS) based on CTA was used to evaluate the extent of thromboembolic vessels [17]. Final infarct volume (FIV) was evaluated based on the low-density areas on NCCT or high-signal areas on MR T2WI or DWI after 2-7 days of follow-up.

#### 2.2.2. Clinical Information Collection

The following baseline information were collected retrospectively: age, gender, atrial fibrillation (AF), hypertension, diabetes mellitus, previous stroke history, smoking history, preoperative plasma BNP level, and the National Institutes of Health Stroke Scale (NIHSS) score. 5 mL of venous blood was collected for laboratory assessment, the blood samples were separated by centrifugation at 4000 r/min for 5 min, and serum BNP concentration was measured immediately in emergency.

The type of stroke was determined based on the Trial of ORG 10172 in Acute Stroke Treatment (TOAST) classification. mRS score of 0-2 indicated good prognosis, and 3-6 indicated poor prognosis. Successful recanalization was defined as the modified thrombolysis in cerebral ischemia (mTICI) grade ≥ 2b.

### 2.3. Statistical Analysis

Nonnormal quantitative data were expressed as the median (interquartile range, IQR), and the Mann-Whitney *U* test was used to examine the difference. Qualitative data were expressed as count (percentage), and the chi-square tests were used to detect the differences. The risk factors of CE were analyzed by logistic regression. Receiver operating characteristic (ROC) analysis was conducted, and the area under the ROC (AUC) was used to assess the value of different parameters (BNP and imaging parameters) on distinguishing CE stroke from non-CE stroke. The relationships between BNP and imaging parameters were assessed using the Spearman correlation analysis. *p* < 0.05 were considered significant. All statistical analysis was performed with SPSS software (version 25.0; SPSS, Chicago, IL) and MedCalc software (version 19.0, MedCalc).

## 3. Results

### 3.1. Baseline Characteristics

The data of 160 patients, including 93 males (58.12%) and 67 females (41.88%), were collected. On admission, compared with the non-CE group, the CE group included older patients and more female patients, and presented lower baseline NIHSS scores, lower incidences of smoking and diabetes mellitus, higher incidence of atrial fibrillation, larger IC volume and IP volume, lower MMR, worse collateral circulation, lower PLT, higher BNP, D-dimer and troponin, shorter onset-to-imaging time and puncture-to-recanalization time, and worse prognosis (all, *p* < 0.05, Table 1).

### 3.2. Risk Factors of CE Stroke

BNP (odds ratio (OR) = 1.004; 95% CI, 1.001-1.009; *p* = 0.038), MMR (OR = 0.736; 95% CI, 0.573-0.945; *p* = 0.016), NIHSS (OR = 1.150; 95% CI, 1.022-1.294; *p* = 0.020), and AF (odds ratio = 556.968; 95% CI, 51.739-5995.765; *p* < 0.001) were the independent predictive factors of CE stroke. It indicated that MMR had higher predictive value compared with other imaging indicators (Table 2).

### 3.3. Diagnostic Performance of Different Markers for CE Stroke

**Figure 2:**
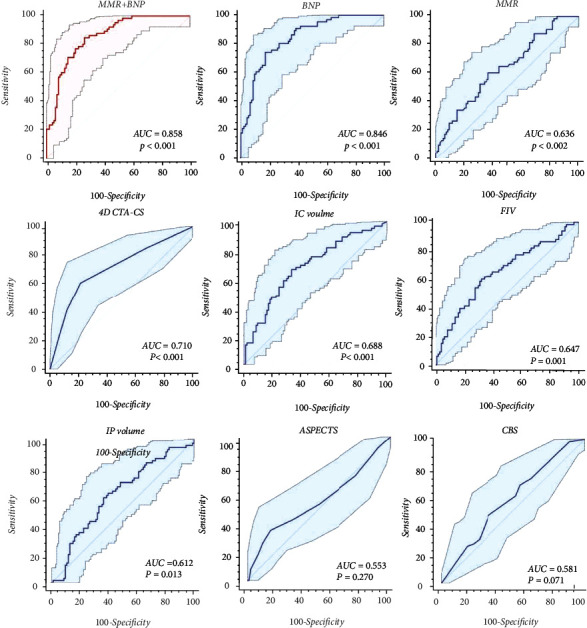
ROC curves for different parameters in predicting CE stroke versus non-CE stroke.

The area under the curves (AUCs) of BNP and MMR were 0.846 (95% CI (0.780-0.898), *p* < 0.001) and 0.636 (95% CI (0.633-0.779), *p* < 0.001), respectively. The cut-off value of BNP was 249.23 pg/mL (sensitivity of 74.24%, specificity of 82.98%) and that of MMR was 2.75 (sensitivity of 60.61%, specificity of 62.77%). Because MMR had a higher predictive value compared with other imaging indicators as shown in Table 2, we selected MMR to be the combined score. The combination of BNP with MMR improved the predictive value. The AUC of this combination was 0.858 (sensitivity of 84.85%, specificity of 73.40%) (Table 3 and Figure 2).

Patients were divided into two groups according to the cut-off value: BNP > 249.23 pg/mL group and ≤249.23 pg/mL group. Regarding 4D CTA-CS, IC, IP, MMR, and FIV, there were significant differences between the two groups (all, *p* < 0.001) (Table 4).

### 3.4. Correlation between BNP and Imaging Parameters

4D CTA-CS (*r* = −0.500, *p* < 0.001), MMR (*r* = −0.461, *p* < 0.001), and CBS (*r* = −0.170, *p* = 0.031) showed negative correlations with BNP. But FIV (*r* = 0.350, *p* < 0.001), IP volume (*r* = 0.276, *p* = 0.026), and IC volume (*r* = 0.361, *p* < 0.001) showed positive correlations with BNP.

## 4. Discussion

Stroke is the leading cause of death worldwide. With the deepening of aging and the increase of risk factors, the burden of stroke in China continues to increase. Therefore, the treatment and monitoring of AIS are very important [18, 19]. EVT is an important therapy for AIS patients with intracranial large vessel occlusion. Since the risk factors, clinical characteristics, and prognostic outcomes are varied in different stroke subtypes, it is essential to determine stroke subtypes to optimize and improve the efficacy of EVT [20, 21]. This single-center retrospective study is aimed at finding the markers that can distinguish CE stroke from non-CE stroke, helping the early diagnosis of AIS patients who need EVT. The major findings of this study were as follows: (1) the BNP level in patients with CE stroke was significantly higher than that in non-CE stroke patients; (2) after adjusting the factors such as gender, age, CHD, and AF, BNP could be used as a blood marker for distinguishing CE stroke from non-CE stroke and MMR could be used as an imaging marker for distinguishing CE stroke from non-CE stroke; (3) the combination of BNP and MMR could improve the predictive accuracy of CE stroke (AUC = 0.858); and (4) there was a correlation between BNP and some imaging parameters including 4D CTA-CS, CBS, MMR, FIV, IP, and IC.

As a biomarker of cardiovascular and cerebrovascular diseases, BNP is a peptide hormone mainly secreted by the heart, which possesses the functions of natriuretic, diuretic, vasodilator, hypotension, antagonizing renin-angiotensin-aldosterone, and inhibiting sympathetic excitation [22]. As a biomarker, BNP is widely used in the diagnosis, stratification, and prognosis of heart failure. In recent years, the application study of BNP in cerebrovascular diseases has increased. Rost et al. have shown that BNP > 140.0 pg/mL is an indicator of CE stroke [8]. Angelantonio et al. revealed that BNP can be used to screen out CE stroke patients caused by thrombus shedding from the left atrium, with the median BNP level of 886 pg/mL [10]. Nevertheless, the previous studies still have some shortcomings. For instance, these studies are mostly carried out in the neurology ward, so most of the patients were in relatively stable conditions but not in the onset of AIS. In these studies, BNP samples were often collected from patients several days after admission. As we have known, BNP level may change over time after the onset of AIS; thus, the onset time of patient enrollment is critical. This study analyzed the 160 AIS patients who were hospitalized in emergency and were required with EVT. The results demonstrated that compared with other stroke subtypes, CE stroke was associated with a higher level of BNP at emergency admission. BNP served as an independent predictor of CE stroke. A level of BNP higher than 249 pg/mL was an indicator of CE stroke, with the sensitivity of 93% and the specificity of 75%.

The results of recent randomized clinical trials on EVT have been accepted in imaging screening criteria, and the importance of imaging examination is also mentioned in the 2018 AHA/ASA guideline [23]. Hence, another focus of this study was to find imaging markers that can distinguish CE stroke from non-CE stroke. This study revealed that MMR was also an independent predictor of CE stroke, which indicates the conditions of cerebral collaterals. The primary collateral circulation, the circle of Willis, is innate and its opening speed is relatively rapid, while the formation and opening of secondary and tertiary collaterals take time [24]. CE stroke is induced by the falling of thrombus formed in the heart under certain conditions, which circulates through the blood to the intracranial artery, resulting in vascular obstruction [25, 26]. This process occurs rapidly, so there is a barely limited time for the formation of the secondary and tertiary collateral circulation of the brain [26]. Patients with CE stroke have poor collateral circulation. The blood perfusion is not enough to maintain the needs of cell physiological activities, resulting in a large area of hypoperfusion [27, 28]. MMR is obtained by the ratio of low perfusion volume to infarct core volume. Therefore, compared with other imaging parameters, MMR can reflect the brain tissue state before EVT more comprehensively and quantitatively. Moreover, Spearman analysis unveiled when the BNP was higher, the value of MMR was smaller, the IC volume and IP volume were larger, the collateral circulation was worse, and the FIV was larger. This may be due to that (1) the lesions of stroke can involve the caudate nucleus, lenticular nucleus, medulla oblongata, and hypothalamus. The secretion of neurogenic BNP is stimulated by ischemia and hypoxia. In the state of cerebral ischemia, the permeability of hematuria barrier is increased, so neurogenic BNP can enter the blood through the abnormal blood-brain barrier. The more severe the ischemic injury of brain tissue is, the higher the plasma BNP level is [29]. (2) The vasodilation effect of BNP can lead to the decrease of peripheral vascular tension, the decrease of blood pressure, and the reduction of cerebral perfusion, resulting in the tissue necrosis of the ischemic penumbra around the infarction [30]. Meanwhile, the promoting effect of BNP on natriuresis also decreases the serum sodium ion level, blood pressure, and blood volume and then further aggravates ischemia [30, 31].

In addition, this study further analyzed the screening value of BNP combined with MMR in patients with CE stroke. The results showed that although the specificity of BNP combined with MMR in screening CE stroke was decreased, the sensitivity and predictive value were significantly improved. CE stroke patients were more serious and had worse prognosis than other subtypes, and it is necessary to determine whether it is CE stroke or not and make the individualized treatment [3, 4]. This study suggested that BNP could be used to quickly screen out patients with CE stroke. MMR combined with BNP may improve the specificity for CE diagnosis, and then, treatment schemes of CE patients can be made in time.

This study still has deficiencies. Firstly, this study is a single-center clinical study and the sample size is small. Secondly, this study is a retrospective study and lacks a randomized control group, so there may exist a selection bias. Further large-scale, prospective, and randomized controlled multicenter clinical studies are needed to verify our findings.

## 5. Conclusion

Our study demonstrates that BNP combined with MMR is valuable for the risk prediction of CE stroke, which will promote the further screening of the high-risk patients with CE stroke and provide more diagnostic information for clinicians.

## Figures and Tables

**Table 1:** Patient characteristics at baseline.

Characteristic	All patients (*n* = 160)	CE stroke (*n* = 66)	Non-CE stroke (*n* = 94)	*p* value
Age, y; median (IQR)	74.50 (62.00, 83.00)	80.00 (69.5, 86.00)	71.00 (59.75, 80.00)	<0.001^∗^
Male, *n* (%)	93 (58.13)	31 (46.97)	62 (65.96)	0.017^∗^
NIHSS, median (IQR)	12.00 (7.00, 17.00)	15.00 (10.00, 21.00)	10.00 (6.00, 14.00)	<0.001^∗^
SBP	145.00 (133.00, 158.75)	147.00 (134.75, 147.00)	143.00 (130.00, 154.00)	0.083
DBP	80.00 (72.00, 90.00)	80.50 (71.75, 94.25)	80.00 (71.75, 88.25)	0.328
Risk factors, *n* (%)				
Smoking	52 (32.5)	15 (22.73)	37 (39.36)	0.027^∗^
AF	63 (39.38)	58 (87.88)	5 (5.32)	<0.001^∗^
Hypertension	125 (78.13)	54 (81.82)	71 (75.55)	0.344
Diabetes mellitus	66 (41.25)	20 (30.30)	46 (48.93)	0.018^∗^
Hyperlipidemia	73 (45.63)	29 (43.94)	44 (46.81)	0.720
CHD	68 (42.5)	32 (48.48)	36 (38.29)	0.199
Previous stroke	73 (45.63)	29 (43.94)	44 (46.81)	0.720
Imaging examinations				
IC volume, mL; median (IQR)	22.55 (8.92, 59.38)	42.14 (18.02, 87.30)	15.27 (5.69, 39.19)	<0.001^∗^
IP volume, mL; median (IQR)	82.24 (43.51, 126.20)	91.43 (54.19, 155.59)	71.14 (35.00, 113.14)	0.016^∗^
MMR, median (IQR)	3.04 (1.67, 6.17)	2.28 (1.18, 4.44)	3.37 (1.95, 7.34)	0.003^∗^
FIV, mL; median (IQR)	38.52 (11.93, 105.32)	61.13 (18.45, 173.46)	26.85 (9.50, 71.56)	0.002^∗^
ASPECTS, median (IQR)	7.00 (6.00, 8.00)	7.00 (4.00, 9.00)	7.50 (6.00, 8.25)	0.253
4D CTA-CS scores, median (IQR)	3.00 (2.00, 4.00)	2.00 (1.00, 3.00)	3.00 (3.00, 4.00)	<0.001^∗^
CBS, median (IQR)	6.00 (3.00, 9.00)	6.00 (0.75, 9.00)	6.00 (3.00, 9.00)	0.077
Thrombus location, *n* (%)				0.089
ICA	47 (29.38)	19 (28.79)	28 (29.79)	
Segment M1	60 (37.5)	28 (42.42)	32 (34.04)	
Segment M2	32 (20.00)	16 (24.24)	16 (17.02)	
A1	8 (5.00)	1 (1.51)	7 (7.45)	
Tandem occlusion	13 (8.12)	2 (3.03)	11 (11.70)	
Laboratory parameters				
Glucose, mmol/L; median (IQR)	7.75 (6.20, 9.68)	7.45 (6.30, 8.43)	7.75 (6.10, 10.93)	0.218
Total protein, g/L; median (IQR)	67.00 (62.00, 71.00)	41.00 (38.00, 45.00)	42.00 (37.00, 45.25)	0.968
Creatinine, *μ*mol/L; median (IQR)	77.50 (65.00, 89.75)	76.00 (66.00, 88.50)	79.00 (64.00, 94.50)	0.733
Urea, mmol/L; median (IQR)	5.68 (4.27, 7.27)	5.74 (4.35, 7.34)	5.68 (4.25, 7.27)	0.770
Uric acid, mmol/L; median (IQR)	327.50 (252.50, 414.00)	347.00 (286.50, 426.00)	310.00 (243.75, 410.25)	0.069
Sodium, mmol/L; median (IQR)	140.10 (138.50, 141.98)	140.00 (138.58, 141.13)	140.40 (138.43, 142.73)	0.259
Potassium, mmol/L; median (IQR)	4.00 (3.70, 4.30)	4.05 (3.68, 4.30)	4.00 (3.80, 4.23)	0.798
D-dimer; median (IQR)	258.00 (137.50, 629.75)	431.00 (205.50, 851.25)	239.00 (88.75, 516.75)	0.002^∗^
PT, s; median (IQR)	11.20 (10.53, 12.10)	11.30 (10.80, 12.43)	11.10 (10.50, 12.00)	0.057
APPT, s; median (IQR)	32.65 (29.85, 35.80)	32.45 (30.45, 36.00)	32.65 (29.70, 35.33)	0.416
Fibrinogen, g/L; median (IQR)	3.07 (2.67, 3.66)	3.07 (2.69, 3.62)	3.07 (2.67, 3.74)	0.997
INR; median (IQR)	0.97 (0.92, 1.05)	0.98 (0.94, 1.08)	0.97 (0.91, 1.04)	0.066
RBC; median (IQR)	4.47 (3.99, 4.86)	4.45 (3.98, 4.84)	4.51 (4.00, 4.88)	0.667
WBC; median (IQR)	8.10 (6.38, 10.17)	7.74 (6.06, 9.62)	8.49 (6.78, 10.43)	0.072
PLT; median (IQR)	196.00 (157.00, 231.00)	178.00 (144.50, 207.50)	204.00 (174.75, 256.50)	<0.001^∗^
BNP, median (IQR)	205.86 (60.94, 443.49)	441.64 (230.24, 642.10)	97.59 (28.42, 223.31)	<0.001^∗^
Troponin; median (IQR)	0.01 (0.00, 0.03)	0.02 (0.01, 0.03)	0.01 (0.00, 0.02)	<0.001^∗^
Time, min; median (IQR)				
Onset to imaging	251.50 (133.00, 463.50)	171.50 (110.50, 307.00)	300.00 (170.00, 595.50)	<0.001^∗^
Imaging to puncture	76.00 (56.25, 104.00)	71.00 (52.00, 102.50)	79.50 (59.50, 109.50)	0.134
Puncture to recanalization	77.50 (51.00, 129.75)	64.00 (44.25, 90.25)	98.00 (57.00, 146.00)	0.001^∗^
Recanalization, *n* (%)	138 (86.25)	55 (83.33)	83 (88.30)	0.369
mRS; median (IQR)	2.00 (0.25, 4.75)	4.00 (1.00, 5.25)	2.00 (0.00, 4.00)	0.003^∗^

CE: cardioembolic; IQR: interquartile range; NIHSS: National Institutes of Health Stroke Scale; SBP: systolic pressure; DBP: diastolic pressure; AF: atrial fibrillation; CHD: coronary heart disease; IC: ischemic core; IP: ischemic penumbra; MMR: mismatch ratio; FIV: final infarct volume; ASPECTS: Alberta Stroke Program Early CT Score; 4D CTA-CS: the modified collateral circulation scoring system on 4D CTA; CBS: clot burden score; ICA: internal carotid artery; M1: M1 segment middle cerebral artery; M2: M2 segment middle cerebral artery; A1: A1 segment anterior cerebral artery; PT: prothrombin time; APTT: activated partial thromboplastin time; INR: activated partial thromboplastin time; RBC: red blood cell; WBC: white blood cell; PLT: platelets; BNP: B-type brain natriuretic peptide; mRS: modified Rankin Scale.

**Table 2:** Multivariate analysis model for CE stroke.

Variables	*β*	SE	Wald *χ*^2^	Exp (B)	Lower 95% CI	Upper 95% CI	*p* value
BNP	0.004	0.002	4.288	1.004	1.001	1.009	0.038^∗^
MMR	-0.306	0.128	5.763	0.736	0.573	0.945	0.016^∗^
4DCTA-CS	0.329	0.368	0.797	1.389	0.675	2.86	0.372
Age	-0.063	0.034	3.459	0.939	0.879	1.003	0.063
Gender	1.602	0.986	2.642	4.963	0.719	34.256	0.104
NIHSS	0.14	0.06	5.372	1.15	1.022	1.294	0.020^∗^
Onset to imaging time	-0.003	0.002	3.797	0.997	0.994	1.000	0.051
CHD	-1.832	0.936	3.828	0.16	0.026	1.003	0.050
FIV	0.000	0.004	0.005	1.000	0.992	1.009	0.946
AF	6.323	1.212	27.194	556.968	51.739	5995.765	<0.001^∗^
Smoking	0.639	0.893	0.512	1.895	0.329	10.897	0.474

CE: cardioembolic; BNP: B-type brain natriuretic peptide; MMR: mismatch ratio; 4D CTA-CS: the modified collateral circulation scoring system on 4D CTA; NIHSS: National Institutes of Health Stroke Scale; CHD: coronary heart disease; FIV: final infarct volume; AF: atrial fibrillation.

**Table 3:** Receiver operating characteristic analysis of BNP and radiology parameters.

	AUC	Sensitivity (%)	Specificity (%)	95% CI	Cut-off value	*p* value
BNP	0.846	74.24	82.98	0.780-0.898	249.23	<0.001^∗^
4D CTA-CS	0.710	60.61	78.72	0.633-0.779	2	<0.001^∗^
IC	0.688	66.67	67.02	0.610-0.759	25.26	<0.001^∗^
IP	0.612	59.09	63.83	0.532-0.688	87.09	0.013^∗^
MMR	0.636	60.61	62.77	0.557-0.711	2.75	0.002^∗^
FIV	0.647	66.67	67.02	0.610-0.759	43.43	<0.001^∗^
ASPECTS	0.553	34.85	84.04	0.472-0.631	5	0.270
CBS	0.581	46.87	67.02	0.500-0.658	4	0.071
BNP+MMR	0.858	84.85	73.40	0.794-0.908	NA	<0.001^∗^

BNP: B-type brain natriuretic peptide; 4D CTA-CS: the modified collateral circulation scoring system on 4D CTA; IC: ischemic core; IP: ischemic penumbra; MMR: mismatch ratio; FIV: final infarct volume; ASPECTS: Alberta Stroke Program Early CT Score; CBS: clot burden score.

**Table 4:** Radiologic data of AIS patients according to the BNP cut-off value.

Variable	BNP		*p* value
	≤249.23 (*n* = 95)	>249.23 (*n* = 65)	
4D CTA-CS	3.00 (3.00, 4.00)	2.00 (0.50, 3.00)	<0.001^∗^
IC	19.45 (7.44, 37.10)	47.29 (13.60, 129.09)	<0.001^∗^
IP	74.15 (35.56, 113.06)	91.36 (51.76, 158.19)	0.016^∗^
MMR	3.31 (1.94, 7.03)	2.60 (1.15, 4.92)	0.031^∗^
FIV	26.70 (9.67, 62.28)	87.01 (20.63, 186.49)	<0.001^∗^
ASPECTS	7.00 (6.00, 8.00)	7.00 (4.00, 9.00)	0.683
CBS	6.00 (4.00, 9.00)	4.00 (7.00, 9.00)	0.102

BNP: B-type brain natriuretic peptide; 4D CTA-CS: the modified collateral circulation scoring system on 4D CTA; IC: ischemic core; IP: ischemic penumbra; MMR: mismatch ratio; FIV: final infarct volume; ASPECTS: Alberta Stroke Program Early CT Score; CBS: clot burden score.

## Data Availability

The data used to support the findings of this study are available from the corresponding authors upon request.
